# Towards a novel influenza vaccine: engineering of hemagglutinin on a platform of adenovirus dodecahedron

**DOI:** 10.1186/1472-6750-13-50

**Published:** 2013-06-16

**Authors:** Antonina Naskalska, Ewa Szolajska, Igor Andreev, Malgorzata Podsiadla, Jadwiga Chroboczek

**Affiliations:** 1 Institute of Biochemistry and Biophysics, PAS, Pawinskiego 5a, 02106 Warsaw, Poland; 2 Institute of Molecular Biology and Genetics, NASU, 150 Zabolotny Str. 143, Kiev, Ukraine; 3 Therex/TIMC/IMAG, UJF-CNRS, UMR 5525, Domaine de la Merci, 38706 La Tronche, France

**Keywords:** Influenza vaccine, Hemagglutinin, Adenovirus dodecahedron, WW-domain adaptor, Vaccine presentation platform, Multivalency

## Abstract

**Background:**

The production process for the current influenza vaccine takes about 6 months and its antigenic composition must be modified annually. In the attempt towards developing influenza vaccine production that would be faster, safer and cheaper we engineered an influenza vaccine in which multiple copies of hemagglutinin (HA) would be delivered by a vector, adenovirus dodecahedron (Ad Dd). Dd is a virus-like particle, formed by assembly of twelve copies of pentameric penton base (Pb) proteins responsible for virus penetration. In order to attach HA to the vector, an adaptor containing WW domains was used. The WW domain is a linear peptide fragment identified as a partner of proline-proline-x-tyrosine (PPxY) motif present at the N-terminal extremity of the Pb protein, which is a building block of Dd. That tandem of three WW domains in fusion with the protein of interest enables interaction with Dd and efficient translocation to the cytoplasm of cells in culture.

**Results:**

Since HA is an oligomeric protein with complicated processing, we prepared six different constructs of HA (A/swan/Poland/467/2006(H5N1)) in fusion with the WW adaptor. Herein we report baculovirus expression and functional analysis of six HA-WW variants. The best behaving variant was successfully delivered into human cells *in vitro*.

**Conclusions:**

Engineering of a soluble complex of HA with Dd, a virus-like particle that serves as a vector, an adjuvant and as a multivalent presentation platform, is an important step toward a novel influenza vaccine.

## Background

The production process for the three influenza vaccines approved by US Food and Drug Administration (FDA) takes about 6 months and their antigenic composition must be modified annually. They are prepared according to a somewhat antiquated technology, known for the last 60 years. The virus is grown in fertilized hens eggs, and the infected allantoic fluid is harvested. The extracted virus is purified, inactivated and treated to produce a vaccine.

Multiple attempts are being described towards developing influenza vaccine production that would be faster, safer and cheaper. These approaches are based on subunit vaccines obtained through expression in heterologous systems. Various prototypes produced in a baculovirus-insect cell expression system have proven safe and effective in clinical studies [1-6]. All subunit influenza vaccines contain the influenza surface glycoprotein, HA, one of the major protein constituents of the influenza virus. HA induces the formation of neutralizing antibodies, creating a first blockage against viral infection [7,8] and it appears that protection provided by the trivalent influenza virus vaccine is mediated primarily by anti-HA neutralizing antibodies [7-9]. Influenza-infected cells produce the HA precursor, HA0, that trimerizes in the endoplasmic reticulum and is transferred through the Golgi apparatus to the cell surface. Proteolytic cleavage generates a trimeric HA protein, each monomer of which consists of two disulfide-linked polypeptides: HA1 that contains the receptor binding- and major antigenic sites, and HA2 containing the fusion peptide. Both cleaved and uncleaved forms of HA exhibit receptor-binding activity, but cleavage is an absolute requirement for membrane fusion activity. Molecular weight of HA monomer ranges from 70 to 84 kDa, depending on influenza virus serotype ([10] and references therein).

The final aim of our project is construction of a vaccine complex that contains influenza antigens delivered by adenovirus dodecahedron (Dd) acting as a vector, adjuvant and a multivalent presentation platform. Adenovirus Dd is a virus-like particle, formed by spontaneous assembly of twelve adenovirus serotype 3 (Ad3) penton base (Pb) proteins responsible for virus penetration [11]. Dodecahedra are devoid of genetic information, and form a multivalent platform where the antigen can be presented in multiple copies; their utility as delivery platform for vaccinal antigens has already been shown [12,13]. In this communication we describe the vaccine component containing the HA antigen.

In order to attach HA to the vector, an adaptor containing so-called WW domains is here used. WW domain, a fragment of 23–35 amino acids flanked by two tryptophans (W), is a partner of the proline-proline-x-tyrosine (PPxY) motif present at the N-terminal extremity of the penton base protein, Dd building block [14]. A tandem of three WW domains, when cloned in fusion with the protein of interest, acts as an adaptor attaching this protein to Dd, which permits its efficient translocation into the cytoplasm of cells in culture [15]. We have already used the WW linker in order to attach an antigen to the dodecahedron, which resulted in massive transfer of the noncovalent complex of vector with the antigen to dendritic cells [12]. Furthermore, intracellular Dd induced activation of dendritic cells. Finally, the Dd carrying antigen underwent efficient capture, processing and presentation by the dendritic cells (op.cit.).

Herein we report baculovirus expression and functional analysis of six HA-WW variants in fusion with the WW adaptor. The best behaving variant was successfully delivered into human cells *in vitro*. Engineering of a soluble complex of HA with Dd, a virus-like particle that serves as a vector, an adjuvant and as a multivalent presentation platform, is an important step toward a novel influenza vaccine.

## Methods

### Hemadsorption

HF cells expressing HAWW and WWHA proteins corresponding to 200 μl culture were suspended in PBS, distributed to wells of the 96-well microtiter plate and sequentially diluted with PBS. Next, 10 μl of 1% hen erythrocyte suspension was added to each well and the plate was incubated for 1 h at room temperature. Hemadsorption was observed under light microscope (Olympus CK, magnification 20000 x) and photographs were taken with Olympus C3040 camera.

### Hemagglutination

Aliquots of protein expressing HF cells (500 μl culture) were suspended in 100 μl of hypotonic buffer, lysed by freeze-thawing, dispensed to U-bottom 96-well microtiter plate and diluted with the hypotonic buffer, as described above. Next, 50 μl of 5% suspension of hen erythrocytes was added to each well and the plate was incubated for 1 h at room temperature. Hemagglutination was inspected visually and the plate was photographed.

### Analysis of HA cell surface expression

Aliquots (500 μl culture) of live expressing HF at 48 h post infection were incubated with anti-HA antibody (100 μl, 1:100 in PBS; 1 h at 37°C) followed by incubation with anti-rabbit FITC-labeled antibody (100 μl, 1:250 in PBS; 1 h at 37°C). Simultaneously, similar aliquots of HF cells were treated for 5 min on ice with trypsin (Sigma, 1 μg per 500 μl cell portion) and the reaction was terminated by addition of the Complete protease inhibitor. Portions of approximately 10000 cells were analyzed by flow cytometry on a FACSCalibur (Beckton Dickinson).

### Overlay with dodecahedra

Lysates of expressing HF cells were separated on 10% polyacrylamide gel, with BSA as a negative control and WW protein as a positive control. Proteins were then transferred onto PVDF membrane (Millipore) and incubated for 2 h at RT with Dd preparation (25 μg/ml in 20 mM Tris pH 7, containing 150 mM NaCl, 2 mM EDTA, 5% glycerol) [16]. After 1 h blocking with 5% nonfat dried milk in TBST, the membrane was incubated with anti-Dd antibody (1:40000 in TBST) and anti-rabbit-HRP secondary antibody (1: 100000) and revealed with the ECL reaction. WW control protein was obtained from pGEX-4-T1 bearing *WW 1,2,3 (Rsp5)* gene by expression in bacteria and purification on affinity column, using GST tag as described in ref. [12].

### Interaction of non-denatured HA variants with Dd, in ELISA format

ELISA plate (Nunc) was coated with Dd solution of 100 μg/ml (2.5 μg/well), blocked with 0.3% BSA in PBS (100 μl/well, 1 h, 37°C). HAWW- and WWHA-expressing HF cells from 200 μl culture were pelleted and suspended in HEPES buffer pH 7, serially diluted with the same buffer and placed in wells of 96-well dish. After 1.5 h incubation with gentle shaking at RT, the wells were washed with 0.3% BSA in PBS and anti-HA primary antibody was added (1:100, 50 μl per well). The plate was incubated for 1 h at 37°C and, after rinsing, with the secondary anti-rabbit HRP-conjugated antibody (1:10000, 50 μl/well, 1 h at 37°C). Wells were washed 3 times with 0.3% BSA in PBS and the reaction was revealed with 3,3′,5,5′-tetramethylbenzidine (Sigma-Aldrich), followed by immediate blocking with 1 N HCl. The absorbance was measured at 480 nm using BIO-TEK Synergy HT fluorimeter.

### Internalization of HAWW_5 in complex with Dd

HAWW_5-expressing HF cells were lysed for 5 min at room temperature with Cytobuster (Novagen) (150 μl per 1 × 10^6^ cells). The resultant lysate was incubated with the dodecahedra for 1 h at RT to allow for the formation of the Dd-HAWW_5 complex, and applied onto HeLa cells grown on coverslips. After 60 min internalization, the cells were rinsed with sterile PBS, permeabilized and fixed for 30 min in cold methanol. After another wash, the cells were incubated with 5% BSA in PBS, and then with primary anti-Dd antibody at 1:1000 or anti-HA antibody at 1:100, each for 1 h at 37°C. Texas Red-labelled anti-rabbit antibody was used at 1:250 dilution as the secondary antibody and cell nuclei were stained with DAPI (1 μg/ml, Pierce). The coverslips were attached to slides using Mowiol (Sigma).

## Results

### Protein engineering, cloning, expression and visualization

**Figure 1:**
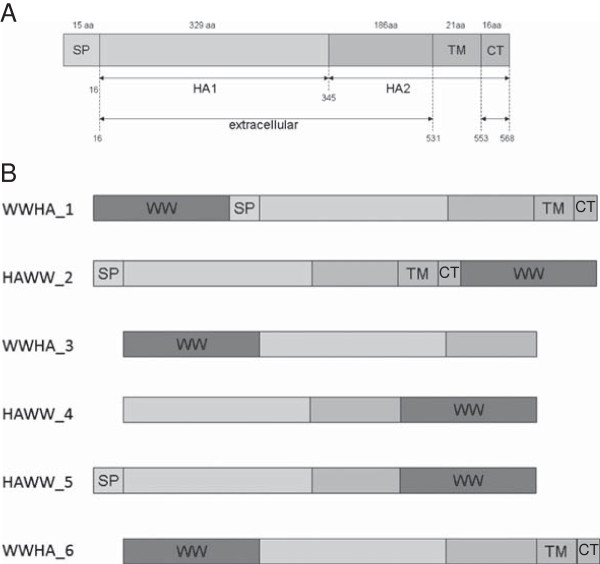
**Hemagglutinin constructs in fusion with WW linker.** (**A**) Schematic representation of HA precursor gene. (**B**). Diagrams of HA constructs. WWHA_1 and WWHA_2 contain the complete HA0 gene. Three constructs have N-terminally and three – C-terminally positioned WW domains. WWHA_3 and HAWW_4 contain HA1 and HA2 domains only, while HAWW_5 is devoid of TM and CT domains, and WWHA_6 is devoid of SP.

In our initial studies on attachment to Dd we used three tandem WW domains of human protein Nedd4 [14]. In order to avoid potential induction of the autoimmune response, we now use WW1,2,3 domains (here called WW) of the yeast Rsp5 protein, that have been shown to have comparable affinity to Dd [12]. Six different constructs of hemagglutinin with N or C-terminally positioned WW domains were prepared (primers are shown in Additional file 1: Table S1). Some clones are devoid of transmembrane (TM) domain and cytoplasmic tail (CT) or/and signal peptide (SP) (Figure 1).

In order to ensure correct folding and oligomerization, recombinant proteins were expressed in the baculovirus system (Additional file 2). Preliminary SDS-PAGE analysis of transfected insect cells confirmed correct selection of positive recombinant clones and successful transfection (Additional file 3: Figure S1), with the titers of recombinant baculovirus ranging from 0.48 to 4 × 10^8^ (Additional file 4: Table S2). Analysis of kinetics of protein expression resulted in setting up the optimal expression time at 48 h post infection. The successful expression of HA variants in HF cells was observed by confocal microscopy (Additional file 5: Figure S3). It has been shown by Li *et al.*[17] that deletion of the transmembrane and cytoplasmic domains of HA might result in secreted HA form. However, only traces of HA proteins were observed in the cell medium of three proteins, HAWW_3, HAWW_4 and HAWW_5, that were devoid of TM and cytoplasmic domains (not shown).

It is known that the mature HA is a glycosylated protein. Two clones, HAWW_2 and HAWW_5, produced proteins that ran as about 15 kDa larger than expected and than other HA proteins (Additional file 3: Figure S1 and Additional file 4: Table S2), which suggested glycosylation. Indeed, when the most interesting protein (see further), HAWW_5, was treated with deglycosylation enzymes, its mobility diminished, indicating removal of glycans (Additional file 6: Figure S4).

### Protein extraction

Four different extraction conditions were tested: sonication, freeze-thawing and incubation with Cytobuster and Proteo Jet. The HAWW_5 protein appeared to be the most soluble variant in all tested extraction methods (lanes 5 in Additional file 7: Figure S2). Proteins WWHA_3 and HAWW_4 showed moderate solubility when extracted by sonication, freeze-thawing or with Proteo Jet lysing solution. WWHA_1, HAWW_2 and WWHA_6, all of which contain transmembrane domains, could not be extracted under any of these conditions. The combination of freeze-thawing and incubation with Cytobuster did not improve this situation (not shown).

### Hemadsorption and hemagglutination

**Figure 2:**
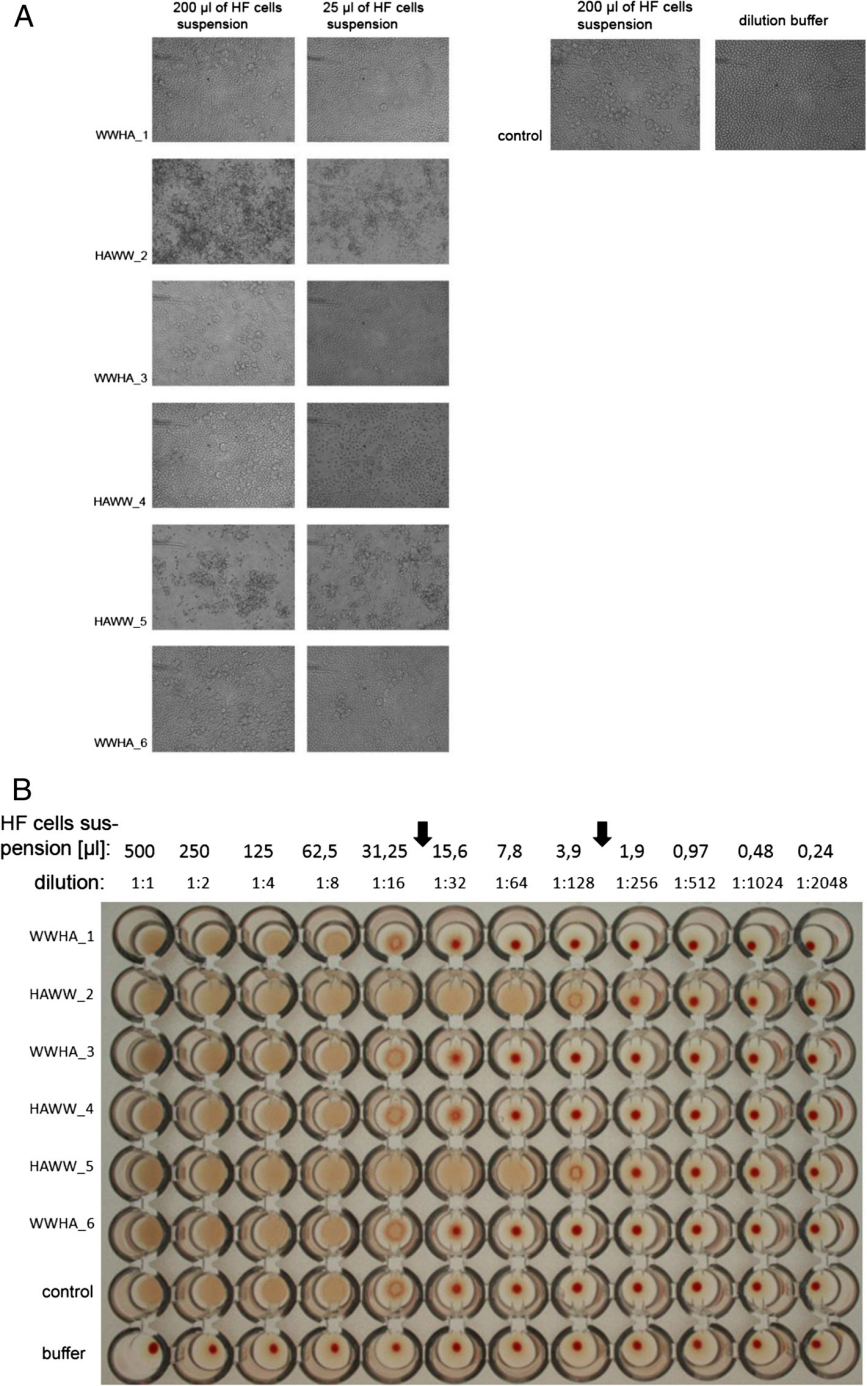
**Functional analysis of HA variants.** (**A**) Hemadsorption assay. Protein expressing HF cells were incubated with a suspension of hen red blood cells and hemadsorption was observed under light microscope. Non-expressing cells and dilution buffer were used as controls. (**B**) Hemagglutination. Lysates of cells expressing proteins were serially diluted and incubated with erythrocytes as described in Materials and Methods. Hemagglutination is shown by a uniform pink suspension of erythrocytes, while the lack of hemagglutination is accompanied by appearance of a red dot in the middle of the well, corresponding to sedimented erythrocytes. Non-expressing HF cells and dillution buffer were used as controls. Arrows indicate dilutions where specific hemagglutination was observed.

Whole or lysed HF cells were tested for their ability to adsorb hen erythrocytes (hemadsorption test) and trigger their agglutination. Very apparent hemadsorption was observed for HAWW_2 and HAWW_5 (Figure 2A), suggesting external membrane HA expression for these clones. Hemagglutination – a uniform pink suspension in microtiter plate - was observed for all variants of the recombinant protein as well as for control cells with no HA expression, when cells were diluted at 1:1 to 1:16. However, HAWW_2 and HAWW_5 revealed specific hemagglutination at higher dilutions - 1:32 to 1:128 (Figure 2B, between arrows). This suggests that only cells expressing HAWW_2 and HAWW_5 produced functional HA. However, in view of the different solubility of the six HA variants, this result may also reflect different accessibility of HAWW/WWHA proteins for erythrocytes.

### Localization of expressed proteins in HF cells

**Figure 3:**
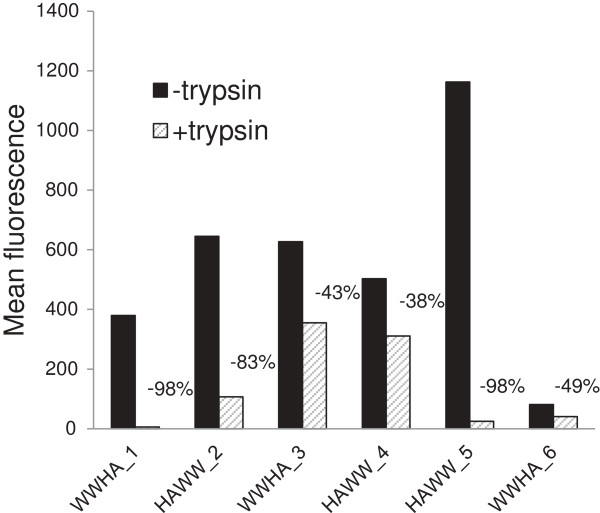
**Surface expression of recombinant proteins, estimated by FACScan analysis.** Black bars show mean fluorescence of live expressing insect cells after incubation with anti-HA antibody. Gray bars represent fluorescence values detected after trypsin treatment. The relative signal decrease (in %) is shown above grey bars.

FACSscan analysis with anti-HA antibody in conjunction with proteolysis was employed to investigate the localization of HAWW and WWHA proteins in living HF cells. In parallel, cells were treated with trypsin and subsequently analyzed by Facscan, in order to analyse protein accessibility to the protease (Figure 3). The HAWW_5 variant appears to be the most abundantly expressed on the insect cell surface and most susceptible to proteolytic removal. Also WWHA_1 and HAWW_2 showed significant signal decrease upon trypsin treatment. It is relevant that together with HAWW_5 these clones contain the signal peptide (SP), known to mediate protein transport to the insect cell surface.

### Interaction with dodecahedron

**Figure 4:**
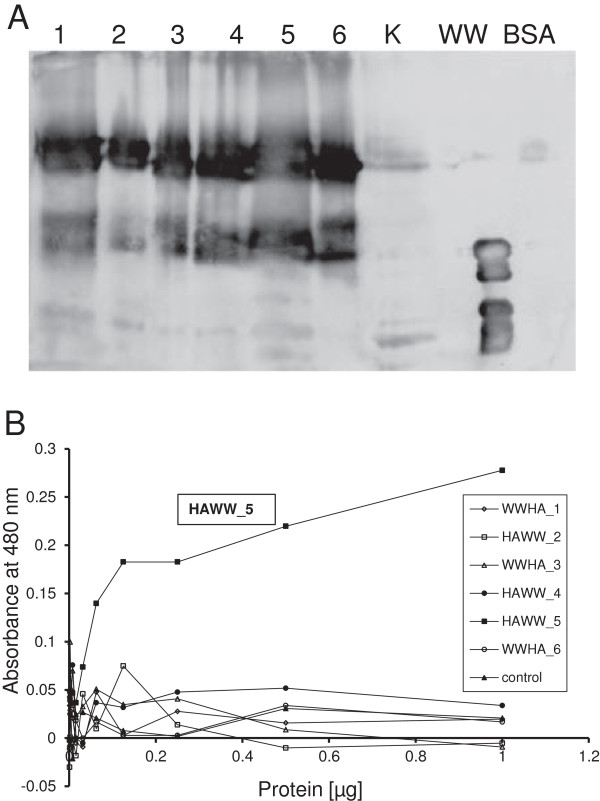
**Interaction of recombinant HAWW/WWHA proteins with Dd.** (**A**) Interaction of denatured HA variants. Proteins were separated by SDS-PAGE, transferred to PVDF membrane and overlaid with the Dd solution. Vector bound to the membrane was detected with specific anti-Dd antibody. Lanes 1 to 6: WWHA_1 to WWHA_6, respectively. K - control HF cells, WW – fragment of Rsp5 protein, containing WW linker (positive control), BSA – bovine serum albumin (negative control). Fusions of HA with WW are denoted with a dot. (**B**) Interaction of non-denatured HA variants. Serial dilutions of HF cells expressing HAWW/WWHA variants were incubated with Dd and the recombinant proteins bound to the vector were detected with anti-HA antibody as described in Materials and Methods.

Proteins containing WW fusions readily interact with dodecahedra [12,13,15]. Since this interaction of WW domains with the PPxY motif of Dd is essential in our vaccine design, we tested HAWW and WWHA proteins for this function. When cell extracts containing SDS-denatured variants of HA were incubated with Dd, all denatured recombinant proteins were able to attach to the vector as detected with anti-Dd antibody (Figure 4A). In the second approach, untreated expressing HF cells were incubated with dodecahedra immobilized in wells of microtiter plate. When formation of a vector–recombinant protein complex was revealed with the anti-HA antibody, only HAWW_5 efficiently bound to dodecahedron (Figure 4B), which confirms the correct folding of this HA fusion variant and its accessibility in live insect cells.

### Internalization of HAWW_5 in complex with Dd in HeLa cells

The analysis of the recombinant HAWW and WWHA proteins permitted selection of one variant, HAWW_5, with an appropriate structure and function (Table 1). In order to demonstrate that the antigen is efficiently internalized through its attachment to the vector, we used confocal microscopy technique (Additional file 8: Figure S5). Analysing vector and antigen co-localization by using different secondary antibodies was not possible due to the rabbit origin of both the anti-Dd and anti-HA antibodies. However, a strong signal obtained with the anti-HA antibody in cells incubated in the presence of the complex compared to lack of the signal in control cells incubated with the same antibody confirmed that the antigen was introduced to cells by the dodecahedric vector. No internalization was observed when cells were incubated with HAWW_5 alone.

**Table 1:** Properties of HA variants

	**WWHA_1**	**HAWW_2**	**WWHA_3**	**HAWW_4**	**HAWW_5**	**WWHA_6**
WW position	N-term	C-term	N-term	C-term	C-term	N-term
Deletion			SP, TM, CT	SP, TM. CT	TM, CT	SP
Solubility			+	+	++	
Hemadsorption		+			+	
Hemagglutination		+			+	
Surface expression	+	++	++	++	+++	-
Interaction with Dd (overlay)	+	+	+	+	+	+
Interaction with Dd (ELISA)	-	-	-	-	+	-

### Co-expression of HAWW_5 with Dd

**Figure 5:**
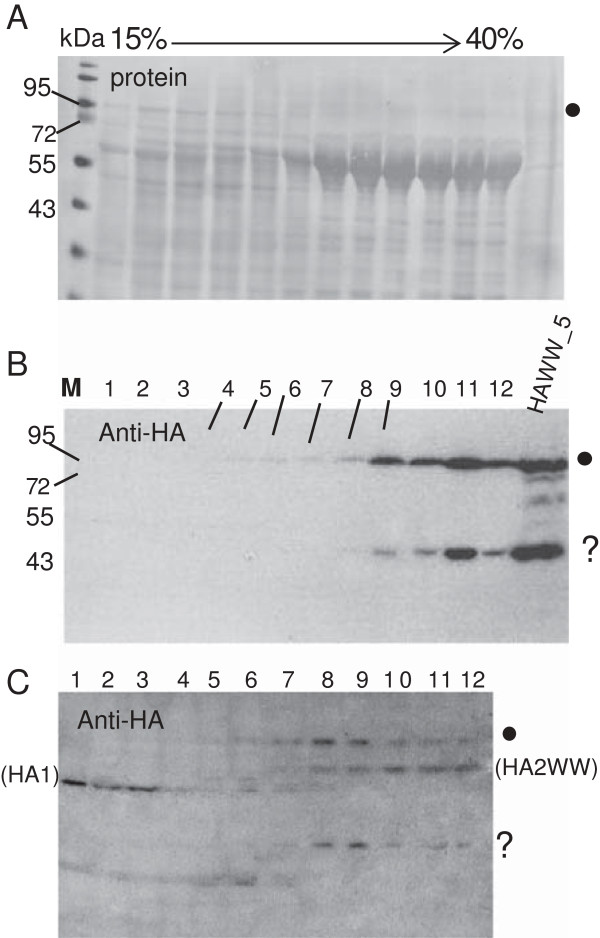
**Formation of soluble Dd-HAWW complexes in insect cells.** (**A**, **B**) HF cells were infected with the baculovirus co-expressing Dd and HAWW_5 at MOI 5 or (**C**) by the baculoviruses expressing Dd and HAWW_5 separatel. After 48 h cells were collected and lysed in hypotonic buffer. Clarified lysates were fractionated on 15–40% sucrose density gradients. Gradient fractions were resolved by SDS-PAGE and transferred onto PVDF membrane. The position of HAWW_5 is indicated by dots. (**A**) Proteins were stained with Ponceau S or (**B**) revealed with anti-HA antibody. M – molecular weight markers, lanes 1 to 12 – gradient fractions. (**C**) Insect cells were infected with the recombinant baculoviruses at a ratio of 1 (Dd) to 10 (HAWW_5). Proteins were analyzed as above by Western blot performed with anti-HA antibody. Full length HAWW_5 is marked with a black dot, tentative HA2WW and HA1 are in brackets and the unknown HAWW fragment is marked with question mark.

Attempts at isolation of significant amounts of HAWW_5 from expressing cells were unsuccessful. Therefore we tried a strategy enabling simultaneous synthesis of Dd and HAWW_5 from a dual-expression baculovirus. This strategy furnished a soluble complex of Dd and HAWW_5, which could be recovered from the heavy fractions of a sucrose density gradient (Figure 5A and B, fractions 7–12). However, the synthesis that occurred at the 1: 1 ratio of appropriate genes led to a large excess of free Dd. We therefore tried the co-expression of the Dd-HAWW_5 complex from two separate baculoviruses, which permitted cell infection at the 10-fold excess of the HAWW_5-expressing baculovirus. The resulting preparation contained much smaller amount of Dd (not shown), with HAWW_5 that localized in the heavy sucrose fractions containing Dd (Figure 5C, fractions 7–12). Approximately a third part of the HA-antibody-reactive material consisted of a full-length HAWW_5. The lower band is most probably HA2WW, as it still migrates in heavy sucrose together with Dd. The HA1, which is devoid of Dd-interacting WW domain, appears in light sucrose (left part of Figure 5C). It is of interest that HAWW_5 undergoes proteolytic processing immediately upon expression, as shown in lane of control HAWW_5 of Figure 5B. Another smaller fragment of HA (marked with question mark) still contains the WW domain, as it localized to heavy sucrose, which indicates its ability to associate with Dd.

## Discussion

Since it has been demonstrated that protection provided by traditional vaccine is mediated primarily by anti-HA neutralizing antibodies [8], it is plausible to think that similar results could be obtained by vaccination with the recombinant HA protein. Therefore, our approach for obtaining induction of humoral influenza immunity consists of construction of a vaccine complex expressed in the baculovirus system that is composed of dodecahedric platform bearing multiple copies of influenza hemagglutinin. In this design, the vector acts as a multivalent delivery platform for antigen presentation as well as an adjuvant. We have previously shown that the complex of Dd with antigen is a potent activator of human myeloid dendritic cells (MDC), and that it is efficiently presented by MDC to antigen-specific CD8+ T lymphocytes [12]. HA protein is here attached externally to the vector through an adaptor containing WW domains recognizing the PY motif present at the N-terminal extremity of Ad3 penton bases that are the Dd building blocks. It is conceivable that use of this multivalent VLP, with remarkable cell penetration ability as well as adjuvant properties, able to present multiple (up to 60) copies of an antigen will permit humoral response achieved at low amount of antigen. It is relevant that the critical function of multimerization of the presentation platform in the immunogenicity of influenza vaccine has already been demonstrated [18]. In addition, symmetric oligomeric VLP formed from the hepatitis B core protein is used as a presentation platform for numerous vaccinal epitopes, including influenza [19].

In the present study we constructed and characterized six variants of HAWW and WWHA proteins. The WWHA_3 variant was not properly folded since it did not display a hemagglutination function, in contrast to the HAWW_2 and HAWW_5 proteins. The main difference seems to be the site of WW linker attachment (N-terminus for WWHA_3 versus C-terminus for HAWW_2 and HAWW_5), which suggests that fusion of the WW domain at the N-terminal position is deleterious to HA folding. It is possible that HAWW_5 protein behaved better than HAWW_2 due to the lack of transmembrane domain TM in HAWW_5. Indeed, only the HAWW_5 protein when non-denatured was able to interact with the vector, showing that it is properly folded, functional and might be a good starting material for influenza vaccine construction. Initially we planned the separate expression and isolation of Dd and HAWW_5 followed by in vitro formation of vaccine complex. However, despite following several published protocols for HA purification, we were unable to recover a significant amount of soluble HAWW_5 protein, conceivable due to the presence of the WW linker.

In the next step, by constructing a baculovirus bearing simultaneously genes for both HAWW_5 and Pb, we attempted to obtain a soluble Dd-HAWW_5 complex directly upon co-expression in insect cells. This resulted in formation of a soluble complex, but with a significant excess of free Dd, suggesting a low occupancy with HAWW_5. Finally, co-expression of two proteins from separate baculoviruses was attempted, in order to be able to manipulate the ratio of infecting baculoviruses. The final soluble complex contained a much smaller amount of Dd. We believe, that this approach might lead to influenza vaccine entirely prepared in cell culture.

## Conclusions

In conclusion, our results suggest the following features of future influenza vaccine:

A) The vaccine should contain an influenza antigen proven to elicit immunity against influenza.

B) The antigen is delivered on a platform of a multivalent, biocompatible and biodegradable vector that was shown to induce maturation of human antigen-presenting cells and thus serve also as immune system adjuvant – hence, there would be no need to use synthetic, potentially harmful adjuvants.

C) The vaccine is produced entirely in insect cells (baculovirus system), which shortens the production cycle and prevents induction of allergic reactions.

Engineering of a soluble complex of HA with Dd, a virus-like particle that serves as a vector, an adjuvant and as a multivalent presentation platform, is an important step toward a novel influenza vaccine. It would make the vaccine production much safer, cheaper and significantly shorten the time necessary for its preparation.

## Abbreviations

Aa: Amino acid residues; CBB: Coomassie brilliant blue; CT: Cytoplasmic domain; HA: Hemagglutinin; HF cells: High-Five, *Trichoplusia ni* cells; MOI: Multiplicity of infection, number of viruses per one cell; Pb: Adenovirus penton base protein; PPxY (PY): Proline-proline-aa-tyrosine motif; Sf21 cells: *Spodoptera frugiperda* cells; SP: Signal peptide; TBST: Tris-buffered saline containing Tween 20; TM: Transmembrane domain; WW: WW1,2,3 (Rsp5), a fragment of the yeast Rsp5 protein containing three WW domains in tandem; VLP: Virus-like particle.

## Competing interests

Authors have no competing interests.

## Authors’ contributions

All authors participated in the conception and design of the study, in the critical revision of the manuscript, and all authors read and approved the final manuscript. AN, IA and MP performed the research (acquisition, analysis and data interpretation), AN, ES and JC drafted the manuscript.

## Supplementary Material

Additional file 1: Table S1 Primers and restriction enzymes used for constructing clones of HA in fusion with WW linker. Click here for file

Additional file 2 Supplementary material. Click here for file

Additional file 3: Figure S1 Analysis of recombinant protein expression. HF cells were infected with the appropriate recombinant baculoviruses, harvested 48 hours post infection, separated on SDS-PAGE and stained with CBB. Lanes 1 to 6: WWHA_1, HAWW_2, WWHA_3, HAWW_4, HAWW_5 and WWHA_6 clones, respectively. K- nonexpressing HF cells. M – molecular weight markers, the values are given in kDa. Click here for file

Additional file 4: Table S2 Analysis of expression. Click here for file

Additional file 5: Figure S3 The successful expression of HA variants in HF cells was observed by confocal microscopy. Click here for file

Additional file 6: Figure S4 Deglycolysation of the recombinant protein WWHA_5. Deglycosylation was carried out as described in Materials and Methods. Deglycosylated (+) and non-treated (-) samples were resolved by SDS-PAGE and analyzed by western blot performed with anti-HA antibody. Click here for file

Additional file 7: Figure S2 Extraction of recombinant proteins. HF cells expressing HAWW and WWHA proteins were incubated with (A) BugBuster, (B) ProteoJet, (C and D) hypotonic Tris buffer and additionally subjected to sonication (C) or freeze/thawing (D). Western blot analysis of supernatants and pellets was performed with anti-HA antibody as described in Materials and Methods. Lanes 1 to 6: WWHA_1 to WWHA_6 clones, respectively. K- control HF cells. Click here for file

Additional file 8: Figure S5 Visualization of the recombinant HAWW and WWHA proteins. Expressing insect cells were analyzed with laser scanning confocal microscopy. Recombinant proteins were detected with anti–HA antibody labeled with Texas Red. Nuclei were stained blue with DAPI. Left-side images show single confocal scans averaged 4 times, whereas Nomarski images are shown on the right. Scale bar corresponds to 10 µm. Click here for file
