# Supplementary material for: Towards a novel influenza vaccine: engineering of hemagglutinin on a platform of adenovirus dodecahedron
Source: BMC Biotechnol. 2013 Jun 16;13:50. doi: 10.1186/1472-6750-13-50 (PMC3688493; doi:10.1186/1472-6750-13-50)
Supplement: Additional file 1: Table S1 — Primers and restriction enzymes used for constructing clones of HA in fusion with WW linker. [file 1472-6750-13-50-S1.docx]

Table 1, Suppl. Mat.

Primers and restriction enzymes used for constructing clones of HA in fusion with WW linker

**Construct Primers Restriction**

**Site**

WWHA F-CAGGG**CATATG**GAGAAAATAGTGC NdeI, SphI

R-AACTC**GCATGC**TTAAATGCAAAT

HAWW F-GGTAAG**CTCGAG**AAATGGAGAAAATAGTGC XhoI, NheI

R-GAAC**GCTAGC**ATAGAAATGCAAATTCTGC

WWHA*(SP^-^TM^-^)*F-CCATCATCGCTAGAC**CATATG**AGTGATCAGATTTGCATTGG NdeI, SphI

R-GCGTT**GCATGC**TATTGGTAGGTTCCTATTG

HA*(SP^-^TM^-^)*WW F-CAGC**CTCGAG**ATGAGTGATCAGATTTGC XhoI, NheI

R-CAGGT**GCTAGC**TGGTAGGTTCCTATTG

HA*(TM^-^)*WW F-GGTAAG**CTCGAG**AAATGGAGAAAATAGTGC XhoI, NheI

R**-**CAGGT**GCTAGC**TGGTAGGTTCCTATTG

WWHA*(SP^-^)* F-CCATCATCGCTAGAC**CATATG**AGTGATCAGATTTGCATTGG NdeI, SphI

R-GCGTT**GCATGC**TATTGGTAGGTTCCTATTG
