# Supplementary material for: Towards a novel influenza vaccine: engineering of hemagglutinin on a platform of adenovirus dodecahedron
Source: BMC Biotechnol. 2013 Jun 16;13:50. doi: 10.1186/1472-6750-13-50 (PMC3688493; doi:10.1186/1472-6750-13-50)
Supplement: Additional file 2 — Supplementary material. [file 1472-6750-13-50-S2.docx]

Supplementary Material

Materials and methods

**Reagents.** The plasmids pGEX-4T-1 (GE Healthcare), pGemEasy (Promega) and pFastBacDual (Invitrogen) were used. Restriction enzymes, ligase and PCR reactants were from Fermentas. The following antibodies were used: anti-HA (National Influenza Center, Lyon), anti-Dd (rabbit polyclonal serum prepared in our laboratory), and the secondary anti-rabbit HRP-conjugated and anti-rabbit Texas Red-conjugated antibodies (both from Jackson ImmunoResearch, Europe). Media used for insect cell cultures of *Spodoptera frugiperda* (Sf) and *Trichoplusia ni* (High-Five, HF) were TC 100 and Express Five, respectively (both from Invitrogen), and for human HeLa cells - EMEM supplemented with 10% fetal bovine serum (FBS) (both from Lonza, Switzerland).

**Protein cloning and expression**.

Recombinant baculoviruses with HA variants. Fragment specifying WW1,2,3 domains (subsequently called WW) from yeast Rsp5 protein and originally provided in pGEX-4T-1, was moved to empty pFastBacDual and cloned under control of p10 promoter. For this, appropriate DNA containing NheI and SphI (PaeI) restriction sites was synthesized by PCR using forward primer: CTTC**GCTAGC**ATTCTTACCCCCTGGTTGG and reverse primer: CACGA**GCATGC**CGCTACATATGGTCTAGCGATGATGG (restriction sites shown in bold) and cloned in NheI and SphI sites of pFastBacDual. HA0 precursor gene (A/swan/Poland/467/2006(H5N1), Epiflu database, accession no EPI156821) was provided in pGemEasy and cloned upstream of the WW insert between XhoI and NheI sites or downstream of the WW insert between NdeI and SphI sites. Six different constructs were designed (Fig. 1) and the appropriate couples of primers were used to synthesize the respective inserts (Table 1, Suppl. Mat.). The PCR products were digested with NdeI and SphI or with XhoI and NheI, respectively, and ligated to linearized WW gene.

Baculoviruses expressing HA antigen and dodecahedron. For simultaneous expression of HA and Dd, pFastBacDualPb plasmid carrying Ad3 wt penton base gene (GenBank acc. no ABB17799.1) under the control of the polyhedrin promoter was used. The sequence coding for HAWW_5 fusion was excised with XhoI and SphI restriction enzymes from pFastBacDual bearing HAWW_5 fusion and ligated to XhoI and SphI linearized pFastBacDualPb, downstream of the p10 promoter. The recombinant plasmids containing HAWW_5 and Ad3 Pb genes were used to prepare baculoviruses expressing simultaneously or separately WWHA and Dd. All plasmids were transfected to competent DH 10 Bac *E. coli* cells. Positive clones were identified by PCR, then the Sf21 cells were transfected with the bacmid DNA following standard Invitrogen protocol. The harvested virus was amplified and the titer of baculoviral stock was determined by plaque assay. For protein expression HF insect cells (2x10^6^ cells/ml) growing in suspension were infected with the recombinant baculoviruses at multiplicity of infection (MOI) 5 and cultured at 27^o^C. Aliquots of each culture taken at different times post-infection were analyzed by Western blot with anti-HA primary antibody at 1:200 and anti-rabbit HRP-conjugated secondary antibody at 1:100 000. The ECL reaction was developed with Sigma reagents, using the Currix 60 AGFA apparatus.

**Protein extraction and analysis.** HF cells expressing HAWW and WWHA fusion proteins were harvested at 48h and suspended in 20mM HEPES buffer, pH 7, supplemented with Complete protease inhibitor (Roche). Aliquots corresponding to 200µl culture were harvested and cell pellets were resuspended in hypotonic buffer (20mM Tris pH 7, containing 50mM NaCl and 2mM EDTA) or Cytobuster lysing solution (Novagen) or Proteo Jet lysis solution (Fermentas). For Bug Buster and Proteo Jet extractions, the suspensions were incubated for 30min at RT, whereas the suspension in hypotonic buffer was divided in two parts and either sonicated or frozen-thawed four times. In some cases three cycles of freezing and thawing of the expressing cell pellets were applied to liberate the recombinant protein. All suspensions were centrifuged and supernatants and pellets were analyzed by Western blot. Proteins were detected with anti-HA antibody. HF cells co-expresing HAWW_5 and Dd were collected at 48h and lysed in hypotonic buffer supplemented with Complete protease inhibitor. Clarified lysates were fractionated on 15–40% sucrose density gradients as described before [17] and analysed by Western blot with anti-HA antibody.

**Analysis of HA expression by confocal microscopy.** Expressing HF cells were harvested 48h post-infection and suspended in HEPES buffer, pH 7, supplemented with Complete protease inhibitor. Aliquots corresponding to 200µl culture were recovered by centrifugation and cells were washed with PBS and fixed with cold methanol for 2h at -20°C. After 3 washes with PBS, cells were placed onto coverslips and left overnight to dry under laminar hood. Coverslips were blocked with 5% BSA in PBS for 45min at RT and then incubated with anti-HA antibody (1:100 in PBS; 1h at 37°C) followed by anti rabbit-Texas Red antibody at 1:1000 in PBS for 1h at 37°C. Nuclei were stained with DAPI (1μg/ml, Pierce) and cells were rinsed again with PBS before mounting the coverslips on slides. The cells were observed with laser scanning confocal microscopy (Nikon) and images were collected with EZ-C1 Nikon CLSM attached to an inverted microscope Eclipse TE2000 E (Nikon) using oil immersion objective x 60. Images show single confocal scans averaged four times, with pixel dwell time of 10µs. Photographs were processed using EZ-C1 Free Viewer software (Nikon).

**Deglycosylation.** Cells expressing HAWW_5 (0.5ml) were suspended in 50µl of denaturing buffer, provided with the New England Biolabs deglycosylation kit, and incubated for 10min at 100^o^C. Next, 3 µl of the Peptide:N-Glycosidase (PNGase), 4µl of 10-fold concentrated reaction buffer and 3µl of NP-40 were added to the reaction mixture and deglycosylation was carried out for 10min. at 37^o^C. The incubation mixture was fractionated by SDS-PAGE and analyzed by Western blot with anti-HA antibody at 1:200 dilution.

Figure legends

Fig. 1. Analysis of recombinant protein expression. HF cells were infected with the appropriate recombinant baculoviruses, harvested 48 h post infection, separated on SDS-PAGE and stained with CBB. Lanes 1 to 6: WWHA_1 to WWHA_6, respectively. K - non-expressing HF cells. M – molecular weight markers, in kDa.

Fig. 2. Extraction of recombinant proteins. HF cells expressing HAWW and WWHA proteins were incubated with (A) Cytobuster, (B) Proteo Jet, (C and D) hypotonic Tris buffer additionally subjected to sonication (C) or freeze/thawing (D). Western blot analysis of supernatants and pellets was performed with anti-HA antibody as described in Suppl. Methods. Lanes 1 to 6: WWHA_1 to WWHA_6 clones, respectively. K - control HF cells.

Fig. 3. Visualization of the recombinant HAWW and WWHA proteins. Expressing insect cells were analyzed with laser scanning confocal microscopy. Recombinant proteins were detected with anti–HA antibody labeled with Texas Red. Nuclei were stained blue with DAPI. Left-side images show single confocal scans, averaged four times, while Nomarski images are shown on the right. Scale bar corresponds to 10 µm.

Fig. 4. Deglycolysation of the recombinant protein WWHA_5. Deglycosylation was carried out as described in Materials and Methods. Non-treated (-) and deglycosylated (+) samples were resolved by SDS-PAGE and analyzed by Western blot performed with anti-HA antibody.

Fig. 5. Internalization of the complex of Dd with HA antigen. Dd-HAWW_5 complex was prepared as described in Materials and Methods. Upper row shows images of control HeLa cells. HAWW and Dd, each separately (middle row), or Dd-HAWW complex (bottom row) were applied to HeLa cells for 1 h. Rabbit anti-HA and anti-Dd antibodies marked red with Texas Red-conjugated secondary antibody were used for antigen and vector detection. Cell nuclei were counterstained with DAPI. Left-side images show single confocal scans, averaged four times, whereas Nomarski images are shown on the right.
