# Supplementary figures and images for: Towards a novel influenza vaccine: engineering of hemagglutinin on a platform of adenovirus dodecahedron
Source: BMC Biotechnol. 2013 Jun 16;13:50. doi: 10.1186/1472-6750-13-50 (PMC3688493; doi:10.1186/1472-6750-13-50)

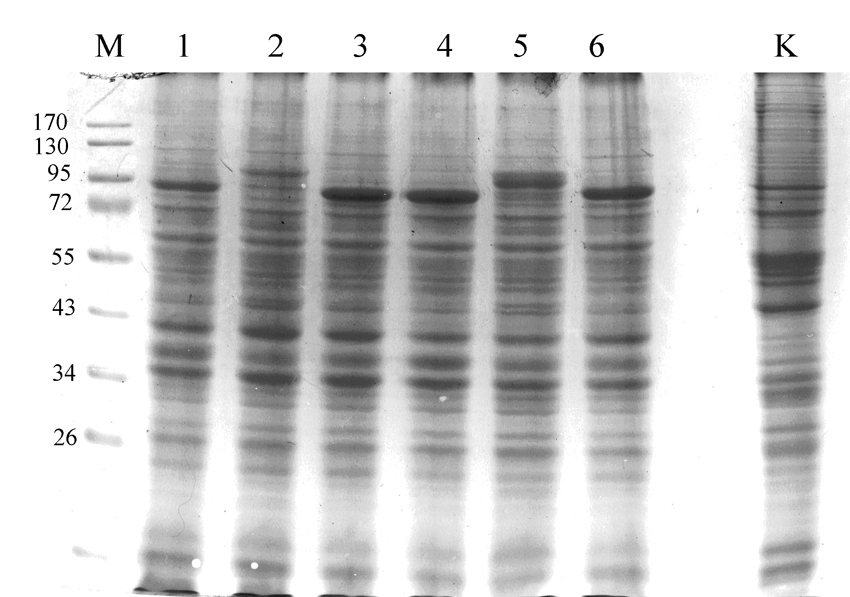


Naskalska et al, Fig 1. Supplementary data

Supplement: Additional file 3: Figure S1 — Analysis of recombinant protein expression. HF cells were infected with the appropriate recombinant baculoviruses, harvested 48 hours post infection, separated on SDS-PAGE and stained with CBB. Lanes 1 to 6: WWHA_1, HAWW_2, WWHA_3, HAWW_4, HAWW_5 and WWHA_6 clones, respectively. K- nonexpressing HF cells. M – molecular weight markers, the values are given in kDa. [file 1472-6750-13-50-S3.docx]

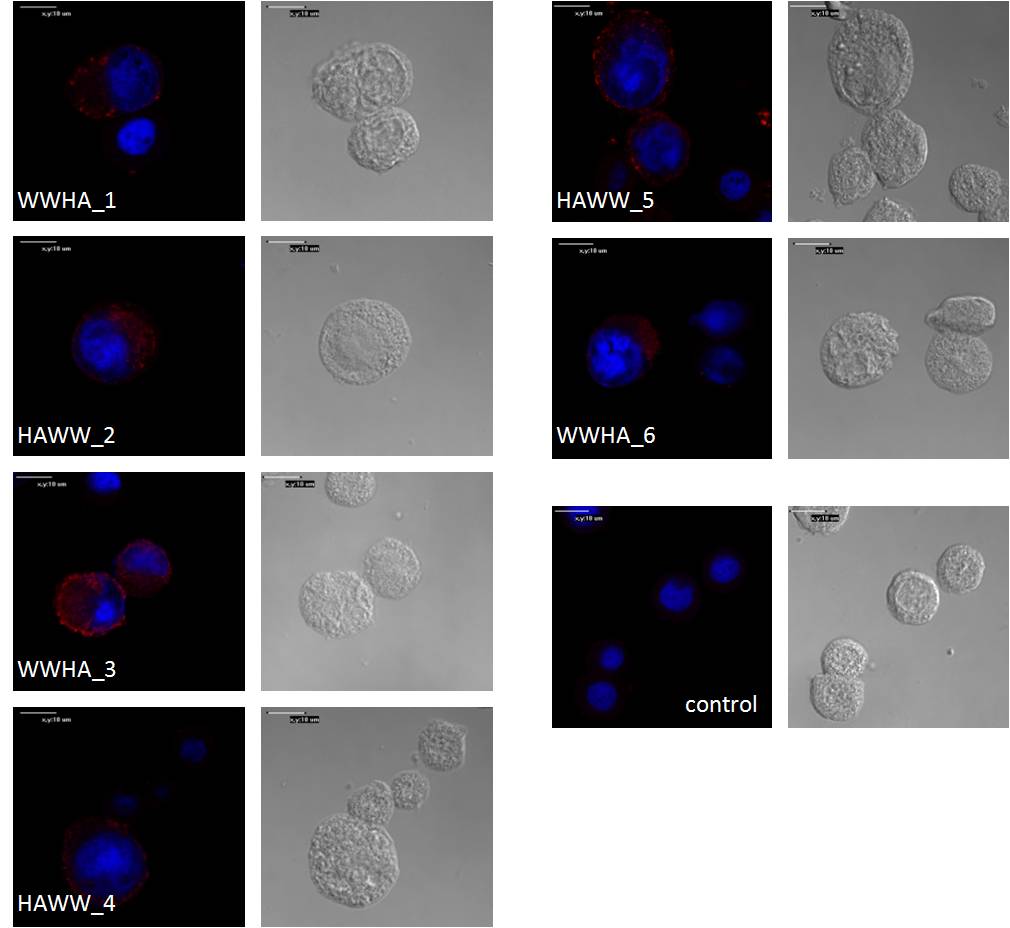


Naskalska et al, Fig 3. Supplementary data

Supplement: Additional file 5: Figure S3 — The successful expression of HA variants in HF cells was observed by confocal microscopy. [file 1472-6750-13-50-S5.docx]

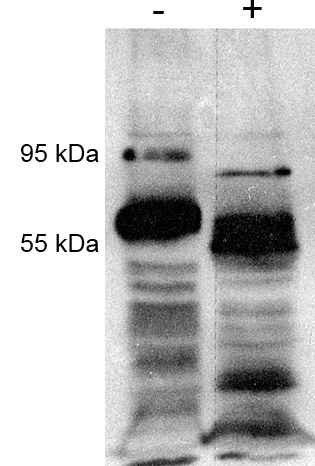


Naskalska et al, Fig 4. Supplementary data

Supplement: Additional file 6: Figure S4 — Deglycolysation of the recombinant protein WWHA_5. Deglycosylation was carried out as described in Materials and Methods. Deglycosylated (+) and non-treated (-) samples were resolved by SDS-PAGE and analyzed by western blot performed with anti-HA antibody. [file 1472-6750-13-50-S6.docx]
