# Supplementary material for: Towards a novel influenza vaccine: engineering of hemagglutinin on a platform of adenovirus dodecahedron
Source: BMC Biotechnol. 2013 Jun 16;13:50. doi: 10.1186/1472-6750-13-50 (PMC3688493; doi:10.1186/1472-6750-13-50)
Supplement: Additional file 4: Table S2 — Analysis of expression. [file 1472-6750-13-50-S4.docx]

Table 2, Suppl. Mat.

Analysis of expression

**Construct Deletion Theoretical Band on Virus titer**

**MW,** kDa **SDS-PAGE,** x10^8^

**MW,** kDa

WWHA1 - 86,7 ~80 0.48

HAWW2 - 86,7 >95 3.25

WWHA3 SP, TM^-^ 81,3 ~80 4.00

HA4WW SP, TM^-^, 81,0 ~80 0.75

HA5WW TM 82,8 >95 3.60

WWHA6 SP 86,0 ~80 0.70
