# Supplementary material for: Towards a novel influenza vaccine: engineering of hemagglutinin on a platform of adenovirus dodecahedron
Source: BMC Biotechnol. 2013 Jun 16;13:50. doi: 10.1186/1472-6750-13-50 (PMC3688493; doi:10.1186/1472-6750-13-50)
Supplement: Additional file 7: Figure S2 — Extraction of recombinant proteins. HF cells expressing HAWW and WWHA proteins were incubated with (A) BugBuster, (B) ProteoJet, (C and D) hypotonic Tris buffer and additionally subjected to sonication (C) or freeze/thawing (D). Western blot analysis of supernatants and pellets was performed with anti-HA antibody as described in Materials and Methods. Lanes 1 to 6: WWHA_1 to WWHA_6 clones, respectively. K- control HF cells. [file 1472-6750-13-50-S7.pptx]

## Slide 1
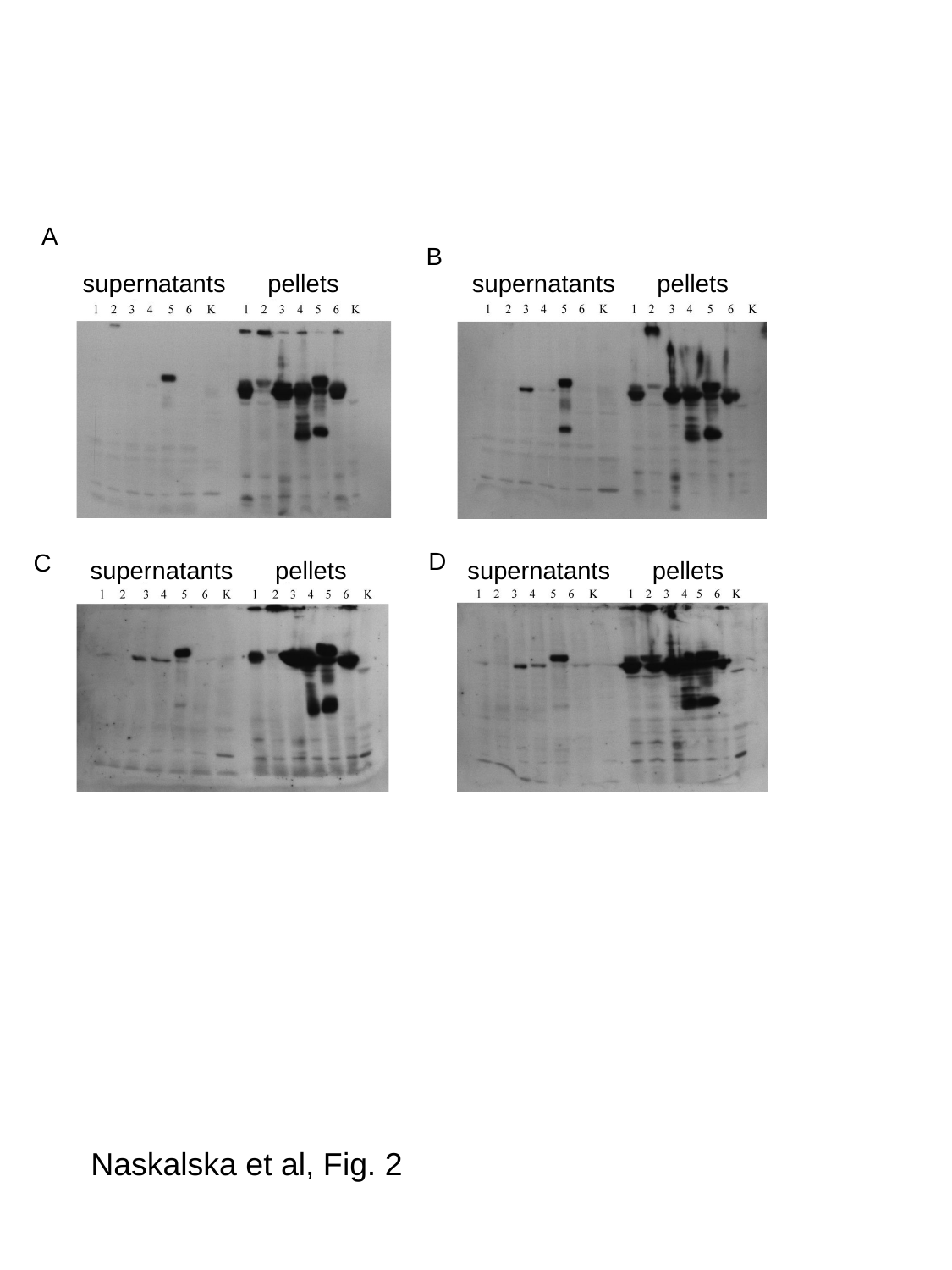

A
B
supernatants pellets
supernatants pellets
D
C
supernatants pellets
supernatants pellets
Naskalska et al, Fig. 2
