# Supplementary material for: Towards a novel influenza vaccine: engineering of hemagglutinin on a platform of adenovirus dodecahedron
Source: BMC Biotechnol. 2013 Jun 16;13:50. doi: 10.1186/1472-6750-13-50 (PMC3688493; doi:10.1186/1472-6750-13-50)
Supplement: Additional file 8: Figure S5 — Visualization of the recombinant HAWW and WWHA proteins. Expressing insect cells were analyzed with laser scanning confocal microscopy. Recombinant proteins were detected with anti–HA antibody labeled with Texas Red. Nuclei were stained blue with DAPI. Left-side images show single confocal scans averaged 4 times, whereas Nomarski images are shown on the right. Scale bar corresponds to 10 µm. [file 1472-6750-13-50-S8.ppt]

## Slide 1
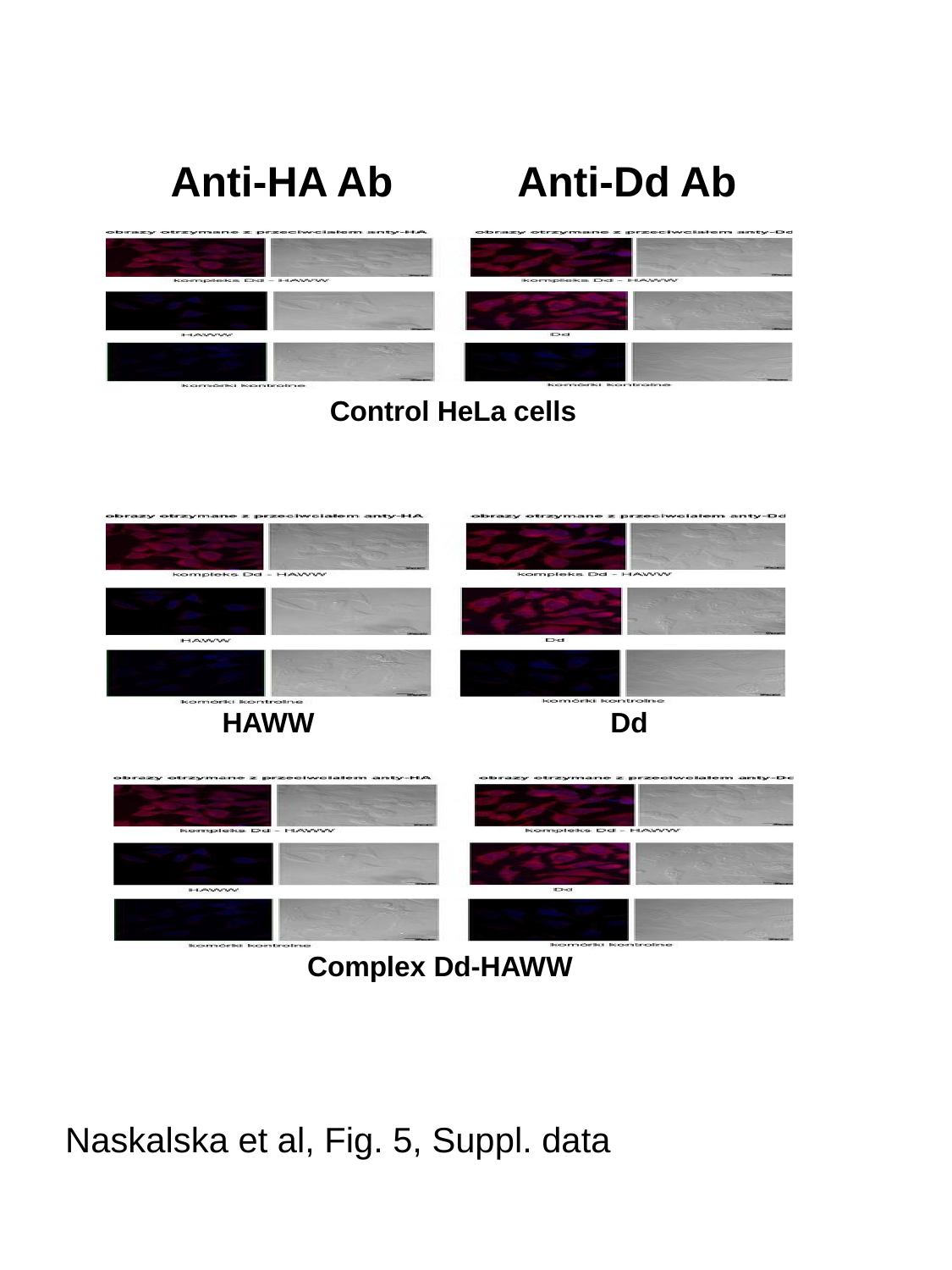

Anti-HA Ab	 Anti-Dd Ab
Control HeLa cells
 HAWW		 Dd
Complex Dd-HAWW
Naskalska et al, Fig. 5, Suppl. data
